# Evaluation of Rat Testicular Cell Populations in Experimental Condition of Diabetes Induced in Early Postnatal Life

**DOI:** 10.3390/cells14211714

**Published:** 2025-10-31

**Authors:** Ekaterina Pavlova, Rosen Ivanov, Desislava Abadjieva, Yordanka Gluhcheva, Emilia Petrova, Ivelin Vladov, Emilia Lakova, Nina Atanassova

**Affiliations:** 1 Institute of Experimental Morphology, Pathology and Anthropology with Museum, Bulgarian Academy of Sciences, Acad. Georgi Bonchev Str., bl.25, 1113 Sofia, Bulgaria; rosen.ivanov@iempam.bas.bg (R.I.); yordanka.gluhcheva@iempam.bas.bg (Y.G.); emilia.petrova@iempam.bas.bg (E.P.); ivelin.vladov@iempam.bas.bg (I.V.); ninaatanassova@bas.bg (N.A.); 2 Institute of Biology and Immunology of Reproduction, Bulgarian Academy of Sciences “Acad. Kiril Bratanov”, 1113 Sofia, Bulgaria; dabadjieva@ibir.bas.bg; 3 Department of Physiology and Pathophysiology, Medical University of Pleven, 5800 Pleven, Bulgaria; tcakova-lakova@mu-pleven.bg

**Keywords:** diabetes mellitus, testis, germ cells, Leydig cells, Sertoli cells

## Abstract

Diabetes mellitus (DM) causes male infertility through the suppression of spermatogenesis and testosterone biosynthesis. The impact of DM on male reproduction has mainly been investigated in adulthood, therefore we aimed to study the developmental effects of DM, induced in early life, on testicular cell population and fertility. Neonatal (NDM) and prepubertal DM (PDM) were induced in immature rats by streptozotocin administration on day 1 or day 10, respectively. Germ (GCs) and somatic cells (Sertoli—SCs and Leydig cells—LCs) were counted in pubertal (25 day) and post-pubertal (45 day) rats in tandem with the measurement of serum testosterone levels and the protein expression of androgen receptor. Glucose levels were higher in PDM than in NDM. Incomplete spermatogenesis and reduced GC number were found in PDM but not in NDM. LC number, testosterone, and luteinizing hormone (LH) levels were differently altered by both types of DM with a pronounced negative impact of PDM. Protein expression of androgen receptor in SCs was altered only in PDM. Reduced sperm concentration and motility was found in both groups. Thus, our results provide new insights into different mechanisms of action of PDM and NDM on developing germ cells that involved disturbances in androgen production by Leydig cells and androgen action in Sertoli cells.

## 1. Introduction

Diabetes mellitus (DM) is a chronic complicated metabolic disorder characterized by hyperglycemia, which often results from defects in insulin secretion, insulin action, or both. According to the International Diabetes Federation, approximately 589 million adults were living with type 1 DM (T1DM) and type 2 (T2DM) in 2024; it estimates that by 2050, this number will increase to 853 million [1]. More than 1.8 million children and adolescents are living with T1DM worldwide, and there is a continuous increase in the number of young patients with T1DM and T2DM [1]. The increased incidence of T1DM has been associated with falling birth rates and compromised fertility [2].

A large number of studies, both in diabetic men and animal models, indicate that DM causes male infertility via action at multiple levels including altered spermatogenesis, degenerative and apoptotic changes in the testes, altered glucose metabolism in Sertoli cells/blood testes barrier, reduced testosterone synthesis and secretion, ejaculatory dysfunction, and reduced libido [3]. In men with chronic diabetes and obesity, late-onset hypogonadism syndrome is more popular. The real obstacles to conducting studies on human male are ethical reasons (e.g., nearly no testicular biopsies were available, therefore no systematic histopathology or molecular data from the human testis of patients with DM are available) [4]. Therefore, the use of animal models has been crucial to provide more detailed molecular and cellular insights into the effect of hyperglycemia on the male reproductive system and fertility.

Many animal studies have employed streptozotocin (STZ)-induced diabetes, as STZ is the most prominent diabetogenic chemical that is widely used in experimental animals for creating animal models of type 1 and type 2 diabetes [5,6]. STZ is a naturally occurring compound, produced by the soil bacterium *Streptomyces achromogenes*, that easily enters the pancreatic beta cells through glucose transporter-2, causing alkylation/damage of the DNA and subsequent cell death resulting in rapid hyperglycemia. STZ has been proven to be a better diabetogenic agent than alloxan, with a wider species effectiveness and greater reproducibility [7]. Moreover, the STZ model mimics many of the acute and chronic complications of human diabetes and provides the established similarities of some of the structural, functional, and biochemical abnormalities to human disease. Hence, it is an appropriate model to assess the mechanisms of diabetes.

The mammalian testis is a complex multicellular organ, separated into two distinct compartments that carry out its principle functions. In the adult testis, spermatogenesis and sperm production occur within the seminiferous tubules, and androgen biosynthesis (steroidogenesis) occurs in Leydig cells located in the interstitium.

Two major events in testis development that occur during sexual maturation are the establishment of spermatogenesis and the development of the adult Leydig cell population responsible for androgen production. Spermatogenesis in mammals is a dynamic and highly regulated process that encompasses numerous proliferating and differentiating steps from spermatogonia to spermatozoa, resulting in the production of male gametes [8].

Androgens are especially important for male sexual differentiation in fetal life, pubertal sexual maturation, and the maintenance of spermatogenesis in adulthood. The effects of androgens are mediated through the androgen receptor (AR), which binds testosterone (T) with high affinity. In the testis, AR is localized in peritubular cells, Leydig cells (LCs), and Sertoli cells (SCs) but not in germ cells (GCs). Preferential action of androgens at stages VII–VIII of the spermatogenic cycle of rats coincided with the maximal expression of AR protein in Sertoli cells, suggesting that androgen support for spermatogenesis is primarily mediated through Sertoli cells [9]—a conclusion that is supported by knockout models for the selective ablation of AR in Sertoli cells (SCARKO mice) [10].

Sertoli cells are involved in the regulation of spermatogenesis, providing nutritional support for germ cells. Glucose metabolism in Sertoli cells produces lactate for germ cells, which is crucial for spermatogenesis, and in particular, it is consumed by pachytene spermatocytes and round spermatids [11]. Germ cells are highly reliant on carbohydrate metabolism as they need energy for their differentiation. However, metabolic stressors, such as diabetes, impair glucose transport and lactate production, compromising energy supply. Chronic hyperglycemia has been shown to increase glucose uptake and reduce lactate production by Sertoli cells, associated with decreased levels of testosterone, luteinizing hormone (LH), and follicle-stimulating hormone (FSH) [12].

Recent reviews have summarized a lot of data on the metabolic and signal pathways of DM action on male reproductive function. The main mechanism of DM is the induction of oxidative stress and inflammation, and in many papers, the role of molecules involved in these pathophysiological processes has been discussed in relation to semen pathology/sperm characteristics and male infertility and profiles of reproductive hormones [12,13,14]. Less data have been published about the effect of DM on the cellular compositions of the testis and their functions. The investigations mainly used experimental models for T1DM induced in adulthood. A limited number of studies utilized neonatally induced DM as a model for T2DM, but the material taken for investigation was from adult animals. Two papers have been published about the effect of DM induced in neonatal or pubertal age (day 15) on developing testis, suggesting that more pronounced alterations occur in early-life induced DM compared with DM induced in adulthood [15,16].

The early postnatal period is crucial for the establishment of spermatogenesis—the resuming of the mitotic division of precursors germ cells on day 4.5 (pre-spermatogonia) gives rise to differentiated spermatogonia followed by the start of meiosis on day 12 in rats [17]. In this respect, our interest was focused on the developmental effect of early DM induced neonatally on day 1 (NDM as it is applied to induce T2DM in adulthood), or prepubertally on day 10 (PDM). Hence, the aim of the present study was to follow the postnatal development of testicular germ and somatic cells (Leydig cells and Sertoli cells) under the condition of experimentally-induced NDM or PDM in relation to androgen production and action. The current work presents a comparative evaluation of the impact of two types of DM, providing new knowledge on the differential effects of early postnatal DM on developing testicular cell populations and the first wave of spermatogenesis. Identification of potential different changes in germ and somatic cells in PDM and NDM rats could contribute to understanding the different response of the testis to hyperglycemia depending on the time of its induction in early life.

## 2. Materials and Methods

### 2.1. Animal Model

The experimental protocol was performed in the Institute of Experimental Morphology, Pathology and Anthropology with Museum, Bulgarian Academy of Sciences according to the ARRIVE guidelines and EU Directive for animal experiments. The study was approved by the Bulgarian Agency for Food Safety, Approval number 282 from 24 September 2020.

Pregnant Wistar rats were purchased from the Experimental and Breeding Base for Laboratory Animals (EBBLA) (Slivnitza, Bulgaria). Mothers were separated into individual standard hard-bottom polypropylene cages, fed a standard diet, and had access to food and water ad libitum. After birth, the pups were i.p. injected with streptozotocin (STZ, Sigma-Aldrich, S0130, St. Louis, MO, USA) at a single dose of 100 mg/kg b.w. (dissolved in ice cold 0.1 M citrate buffer, pH 4.5) on day 1 to induce neonatal diabetes mellitus (NDM) or on day 10 to induce prepubertal diabetes mellitus (PDM) [6,15]. Control age-matched animals were injected with citrate buffer. Two days after STZ injection, blood glucose was measured using a glucometer (Accu-chek Performa, Roshe, Basel, Switzerland). Rats were considered diabetics when their blood glucose levels exceeded 12 mmol/L [15].

At weaning (day 25), the male pups were separated from their mothers and left until the end of the experiment on day 45 or day 65 (for glucose and semen sampling). The experimental animals were divided into three groups (control, NDM and PDM) for the ages investigated. The rats were sacrificed (Small Animal Decapitator, Stoelting™ 51330, Wood Dale, IL, USA) under light anesthesia on postnatal days 25 (puberty) and 45 (post puberty/early adulthood). Blood was obtained after decapitation, and serum was stored at −80 °C for subsequent analysis. Both testes were excised, and the left testis was fixed in Bouin solution, dehydrated, and embedded in paraffin for further histological studies.

Sexually mature 65-day-old rats (control, NDM, and PDM groups) were used for the analysis of sperm concentration and sperm motility.

### 2.2. Measurement of Body and Testis Weight

The body weight of the animals aged 25 and 45 days was measured before sacrifice (n = 10 in each experimental group). Testes of the control and experimental rats were excised, weighed, and the relative testis weight (gonado-somatic index) was calculated as a ratio of the average weight of both testes to body weight, multiplied by 100.

### 2.3. Measurement of Serum Glucose

Glucose levels (non-fasting on day 25 and fasting on days 45 and 65) were evaluated in the sera of control and diabetic animals (n = 10 in each experimental group) using commercial kits (Chema Diagnostica, Monsano, Italy) on a BA-88 biochemical analyzer (Mindray, Shenzhen, China).

### 2.4. Measurement of Serum Testosterone, Luteinizing Hormone, and Insulin

Serum levels of testosterone, luteinizing hormone (LH), and insulin (n = 10 in each experimental group) were evaluated by enzyme-linked immunosorbent assay (ELISA) according to the instructions of the kit manufacturer (Elabscience Biotechnology Co., Ltd., Wuhan, China). Competitive ELISA for testosterone (Cat. No. E-EL-0155) and sandwich-ELISA for luteinizing hormone (Cat. No. E-EL-R0026) and insulin (Cat. No. E-EL-R3034) were performed. The optical density was read at 450 nm on an ELISA Reader BioTek (BioTek Instruments, Winooski, VT, USA). Curve Expert Professional 2.7 software was used. The obtained values were multiplied by the dilution factor (×6 for LH and ×25 for insulin) to calculate the final concentration of the hormones. The concentration of testosterone and insulin is presented in ng/mL and for the luteinizing hormone in mlU/mL.

### 2.5. Stereological Analyses

For morphometric/stereological analyses of germ and Sertoli cells on days 25 and 45, Bouin fixed, 5 µm paraffin embedded tissue sections were stained with hematoxylin and eosin (H&E) (n = 6 in each experimental group). For Leydig cell enumeration, tissue sections were immunostained for 3β-hydroxysteroid dehydrogenase (3β-HSD), which is specific marker for steroid-producing cells. Testicular cell composition was estimated using standard stereological techniques involving the point counting of cell nuclei to determine the nuclear volume per testis, namely Sertoli cells, Leydig cells, and different maturational stages of germ cells, as previously described [18]. In brief, cross-sections of the testes were examined using a 63× objective and a 121-point eyepiece graticule (Leica Microsystems, Wetzlar, Germany) fitted to a Zeiss AxioScope A1 microscope (Zeiss, Oberkochen, Germany). Applying a systematic sampling pattern from a random starting point, 32 microscopic fields (3872 points) were counted for each animal. Points falling over Leydig cells, Sertoli cells, or germ cell nuclei (including spermatogonia, spermatocytes, round and elongated spermatids), seminiferous epithelium, interstitium, and seminiferous tubule lumen were scored and expressed as relative (%) volume per testis. Values for percent nuclear volume were converted to absolute nuclear volume (ANV) per testis by reference to testis volume (=weight) because shrinkage was minimal. Cell nuclear volume can be equated to numbers of cells per testis, assuming no change in nuclear diameter of the target cell in the different experimental groups [10,18].

### 2.6. Immunohistochemistry (IHC) for Androgen Receptor (AR), 3β-Hydroxysteroid Dehydrogenase (3β-HSD), and Testicular Angiotensin Converting Enzyme (tACE)

Unless otherwise stated, all incubations were performed at room temperature. The deparaffinized and rehydrated 5 μm sections of the control and experimental testes from animals aged 25 and 45 days (n = 6 in each experimental group) were subjected to a temperature-induced antigen retrieval step in 0.01 M citrate buffer, pH 6.0—tACE, AR, and 3β-HSD. Endogenous peroxidase activity was blocked by immersing all sections in 3% (*v*/*v*) H_2_O_2_ in methanol for 30 min, followed by two 5-min washes in Tris-buffered saline (TBS). To block nonspecific binding sites, sections were incubated for 30 min with 10% blocking serum in TBS containing 5% bovine serum albumin (BSA): for AR, it was normal swine serum; for tACE and 3β-HSD—normal rabbit serum. After that, primary antibodies were added to the sections at appropriate dilutions in blocking serum and incubated overnight at 4 °C in a humidified chamber. Antibodies were applied as follows: rabbit polyclonal anti-androgen receptor (N-20; sc-816 Santa Cruz Biotechnology, Inc., Dallas, TX, USA), diluted 1:200; goat polyclonal anti-tACE (sc-12187, Santa Cruz Biotechnology, Inc., Dallas, TX, USA), diluted 1:500; goat polyclonal anti-3β-HSD (P-18; sc-30820, Santa Cruz Biotechnology, Inc., Dallas, TX, USA), diluted 1:500. After two 5-min washes in TBS, sections were incubated with biotinylated secondary antibodies as follows: for AR—swine anti-rabbit (dilution 1:500, DAKO Cytomation, Glostrup, Denmark); for tACE and 3β-HSD—rabbit anti-goat (Vector BA-5000) for 30 min. After two additional 5-min washes in TBS, sections were incubated for 30 min with avidin-biotin conjugated to horseradish peroxidase (ABC Reagent, Vector Laboratories Inc., Newark, CA, USA). Sections were washed twice in TBS, and immunostaining was developed using 3,3′-diaminobenzidine (liquid DAB; DAKO Corp., Glostrup, Denmark) The sections were counterstained with hematoxylin, dehydrated before mounting, and observed under a light microscope Zeiss AxioScope A1 (Carl Zeiss, Oberkochen, Germany).

Negative controls were run in parallel by omitting the primary antibody under the same conditions. For AR, the negative control was also conducted by pre-absorption of the primary antibody with peptide immunogen sc-816P.

### 2.7. Assessment of Spermatogenesis by Seminiferous Tubules with tACE Phenotype on Day 45

Completion of the cycle of the seminiferous epithelium was performed by the evaluation of the number of seminiferous tubules (STs) with normal tACE vs. altered phenotype at the early (I–IV), middle (VII–VIII), or late stages (IX–XIV) of the spermatogenic cycle (n = 6 animals in each group). For this purpose, the method for Johnson’s score [19] was applied, and 100 ST were examined across different fields (covering the whole section) using Zeiss AxioStar Plus Microscope (Carl Zeiss, Oberkochen, Germany) at a magnification of ×200 (20× objective). Data are expressed as a percentage of normal or altered ST per all tubules from each stage group.

### 2.8. Quantification of Androgen Receptor Immunostaining Intensity

For quantification of the protein expression (intensity of AR immunostaining) in 25- and 45-day-old rats (n = 6 in each experimental group), we applied a systematic sampling pattern from a random starting point. Each second microscope field of testicular cross section (following vertical direction from the top to the bottom of the tissue cross section) was used to capture the microscope image at a magnification of ×400 (40× objective). Images were obtained through a Zeiss AxioScope A1 light microscope (Carl Zeiss, Oberkochen, Germany) equipped with an AxioCam ERC 5s-Zeiss digital camera (Carl Zeiss, Oberkochen, Germany).

#### ImageJ

For image analysis, RGB images with a resolution 2048 × 1536 pixels saved as .jpg were used. We processed the images using the open source software ImageJ 1.54p, https://imagej.net/ij/ (accessed on 28 October 2025), and its version with multiple plugins—Fiji. The intensity of AR immunostaining was evaluated after color deconvolution using the IHC profile plugin with the selection of Vector “H DAB” and selection of Color_2. AR is localized in the nuclei of Sertoli, perutubular, and Leydig cells in the testis. Our regions of interest (ROIs) were the nuclei of Sertoli cells, which were manually outlined and added to ROI Manager, and the staining intensity was measured as the “mean gray value” parameter. In ImageJ, the pixel intensity values for any color range from 0 to 255, where 0 represents the darkest shade and 255 represents the lightest shade of the color [20]. Based on this, the staining intensities were divided into three groups: strong (between 1 and 85), moderate to weak (between 86 and 170), and faint to negative (between 171 and 255). We presented the values as reciprocal staining intensity (RSI), where RSI = 255—mean gray value. Therefore, the AR staining intensity was defined as strong at RSI values between 171 and 255; moderate to weak between 169 and 86, and faint to negative when the RSI values were between 85 and 0.

For 25-day-old animals, STs were scored until 100 Sertoli cell nuclei per animal were measured.

For each animal aged 45 days, STs from each of three stage groups (I–VI; VII–VIII; IX–XIV) were scored until 100 Sertoli cell nuclei per stage group were measured. In the control group, a comparison in intensity of AR immunostaining was conducted between stages I–VI (early) and VII–VIII (middle) and IX–XIV (late stages). The same approach was applied in NDM animals where the spermatogenic cycle was completed.

Due to incomplete spermatogenic cycle in 45-day-old PDM rats, where a partial or complete lack of elongating spermatids in stages I–VIII was established, it was not possible to distinguish the early (I–VI) from the middle (VII–VIII) stages. For this reason, in the PDM animals, a comparison in intensity of AR immunostaining was performed between stages I–VIII and the late stages (IX–XIV). Differences between experimental groups were evaluated by a comparison of the intensity of AR immunostaining between stages I–VIII and the late stages (IX–XIV).

### 2.9. Sperm Concentration and Sperm Motility

The sperm concentration and motility were evaluated by computer-assisted sperm analysis (SCA^®^, Sperm Class Analyzer, Microptic^®^, Barcelona, Spain). Briefly, the semen samples were collected from both cauda epididymides of 65-day-old control and diabetic rats (NDM and PDM) (n = 10 in each experimental group) and placed in pre-warmed (37 °C) HEPES solution (pH 7.4). After incubation at 37 °C for 20 min, 5 μL of each sample was transferred to a Leja 20 chamber (Leja Products B.V., Nieuw-Vennep, The Netherlands) and analyzed using a microscope (Nikon Eclipse E200, Nikon, Tokyo, Japan) with a 10× objective (Nikon 10×/0.25 Ph1 BM, Nikon) under negative phase contrast and a camera with high resolution (768 × 576 pixels). All samples were evaluated twice to determine the sperm concentration and motility (percentage of sperm moving faster than 10 µm/s).

### 2.10. Statistical Analysis

The obtained data were presented as the mean value ± standard error (SE) and 95% confidence interval for means. The Shapiro–Wilks test was used to test for the normality distribution of data in the animal experimental groups. The mean values were compared with one-way ANOVA including test of homogeneity of variances and Bonferroni or Games–Howell post hoc test, depending on whether equal variances were assumed or not assumed. The comparison of a pair of indicators in each group (two dependent samples) was used with the paired samples test (for AR expression). Statistical analysis was performed using IBM SPSS Statistics (v.25), where statistical significance was considered at *p* < 0.05.

## 3. Results

### 3.1. Serum Glucose Levels and Diabetic Status

Diabetic status on day 25 (puberty), day 45 (post puberty/early adulthood), and day 65 (adulthood) was validated by an increase in serum glucose levels >12 mmol/L [15]. On day 25, the NDM rats were relatively normoglycemic. On day 45, the serum glucose level was insignificantly higher than the control (27%), while the insulin concentration was lower by 28% (control—17.50 ± 3.88 ng/mL vs. NDM 12.58 ± 1.81 ng/mL). PDM animals were hyperglycemic at both ages, though this was more evident on day 45 when the glucose level was elevated by 93% (Table 1) compared to the control, and the serum insulin levels were reduced by 60% (control—17.50 ± 3.88 ng/mL vs. PDM 6.90 ± 1.57 ng/mL). On day 65, the glucose levels were significantly elevated by 80% in both diabetic groups compared to the control.

### 3.2. Body Weight, Absolute, and Relative Testis Weights

**Figure 1:**
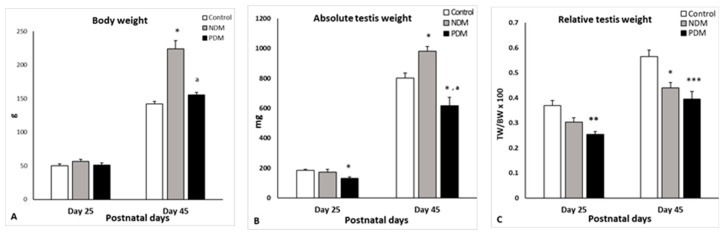
Body weight ((**A**); g), absolute testis weight ((**B**); mg) and relative testis weight [(**C**); testis weight (TW) to body weight (BW) ratio] on days 25 and 45 of the control and diabetic rats. Data represent the mean value ± standard error (SE); * *p* < 0.05; ** *p* < 0.01; *** *p* < 0.001—statistical significance compared to the control value; a—statistically significant difference compared to NDM. NDM—neonatally-induced diabetes; PDM—prepubertally-induced diabetes.

Body weight of the 25-day-old pubertal rats was not changed in PDM and slightly increased in NDM (by 13%). Diabetic rats aged 45 days were heavier than the control animals, and the significant increment in body weight was more pronounced in NDM (>50%) compared to PDM, where the value did not differ significantly than the control (Figure 1A).

The absolute testis weight on day 25 was not affected by NDM. The relative testis weight (gonado-somatic index) was expressed as testis weight to body weight ratio and the value was insignificantly lower than the control (by 18%). However, PDM caused a 30% significant reduction in both the absolute and relative testis weights at this age. By the end of puberty and early adulthood (day 45), NDM caused a significant increase by 20% in absolute testis weight, while the relative testis weight was significantly lower by 20% than the control. In post-pubertal PDM (day 45) animals, both the absolute and relative testis weights were significantly reduced to a similar extent (by 25–30%) compared to the control values (Figure 1B,C).

The absolute volumes of interstitium, seminiferous epithelium, and lumen in NDM and PDM rats on day 25 followed the trend of testis weight. In 45-day-old NDM animals, the volumes of seminiferous epithelium and tubular lumen were increased compared to the control values. The luminal volume was more than 2-fold higher than the control but statistical significance was not reached due to high variation between animals within the NDM group (in three out of six animals, the lumen was expanded). In the PDM group, the three parameters decreased with significance found for seminiferous epithelium (Table 2).

### 3.3. Morphology of the Testis and Assessment of Spermatogenesis. Evaluation of the Seminiferous Tubules with tACE Phenotype

Morphological observation on day 25 revealed the intact histology of testis in both diabetic groups (NDM and PDM). At this age, spermatogenesis proceeds to the stage of late pachytene and diplotene spermatocytes. On day 45, spermatogenesis in the control rats was completed, as evident by the presence of the full complement of germ cells (from spermatogonia to spermatozoa) as well as their associations through all 14 stages of the cycle of the seminiferous epithelium. Although release of spermatozoa into the tubular lumen could be seen in the middle stages (VII–VIII) of the cycle, the rats were not considered as sexually matured as spermatozoa could not be isolated from the cauda epididymis. Morphological changes in the testis of diabetic rats were demonstrated by the visualization of testicular angiotensin-converting enzyme (tACE), known as a specific marker for elongating spermatids (step 8 to step 19 of spermiogenesis), which is expressed in a stage-specific manner. As we previously reported, the low intensity of protein expression appeared in the cytoplasm of elongating spermatids at step 9 in stage IX of the spermatogenic cycle, followed by a gradual increase at the late (XII–XIV) and early stages (I–VI), with maximal expression at stage VII–VIII (in step 19—elongating spermatids, the final step of spermiogenesis) [21].

**Figure 2:**
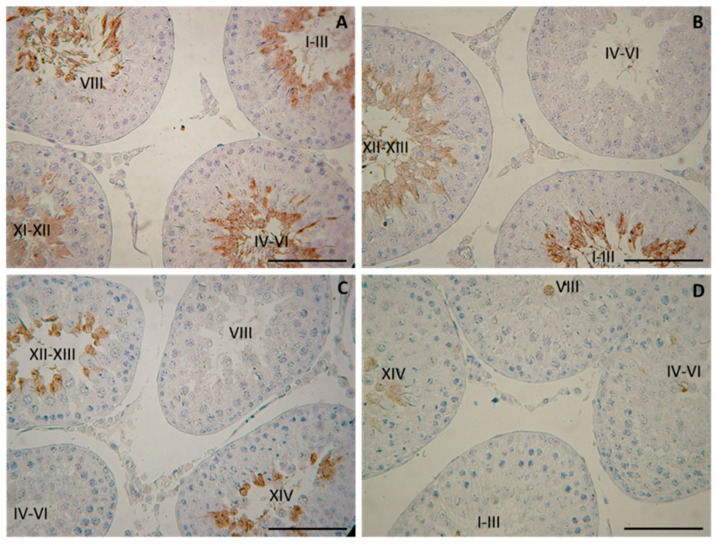
Immunohistochemical localization of testicular angiotensin-converting enzyme (tACE) in the testes of 45-day-old rats—controls (**A**) and prepubertally induced diabetic rats (**B**–**D**). Scale bar = 50 µm.

On day 45, spermatogenesis was completed in the NDM but not in PDM animals where the germ cell complement of the ST was altered. Different degree of delay in spermatid development was observed in PDM rats (Figure 2), which was demonstrated by STs with altered tACE phenotype visualized by a lack of elongated spermatids in stages IV–VI (Figure 2B), or in stages VII–VIII (Figure 2C), or in stages I–III and late stages IX–XIV (Figure 2D). Quantification of STs with altered tACE phenotype showed that all STs in stages IV–VII were lacking in elongated spermatids; 70–97% from STs in stages I–III was devoid of elongated spermatids. STs in late stages IX–XIV were less deprived of elongated spermatids—in four animals, 7–9% STs were deficient in elongated spermatids and in two other animals, 38–65% STs did not contain elongated spermatids.

### 3.4. Quantification of Spermatogenesis. Germ and Sertoli Cell Counts

**Figure 3:**
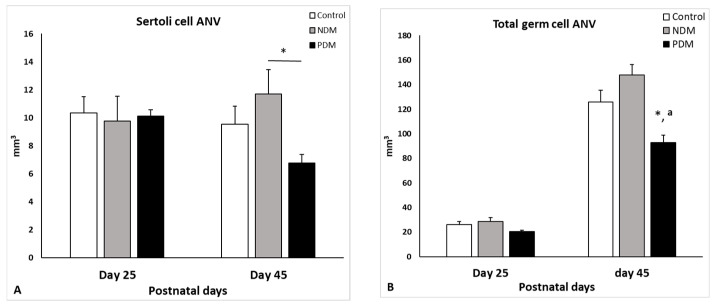
Absolute nuclear volume (ANV, mm^3^) of Sertoli cells (**A**) and total germ cells (**B**) on day 25 and day 45 of control and diabetic rats. Data represent the mean value ± standard error (SE); * *p* < 0.05—statistical significance compared to the control value; a—statistically significant difference compared to NDM. NDM—neonatally-induced diabetes; PDM—prepubertally-induced diabetes.

Seminiferous tubules in the testis comprise Sertoli cells and different types of developing/differentiating gem cells. Our data for the Sertoli cell count revealed that on day 25, the absolute nuclear volume (ANV) in both diabetic groups (NDM and PDM) was not different from the control (Figure 3A). On day 45, the Sertoli cell ANV (SC-ANV) was insignificantly altered by the hyperglycemia—the mean value was higher (by 20%) in the NDM group and lower (by 30%) in the PDM group (Figure 3B).

Total germ cell ANV (TGC-ANV) was calculated as a sum of ANV of spermatogonia, spermatocytes, and spermatids (round spermatids, steps 1–8 and elongating spermatids, steps 9–14). In the controls, the germ cell complement on day 25 comprised spermatogonia and spermatocytes, and on day 45—spermatogonia, spermatocytes, and spermatids. The TGC-ANV was insignificantly increased (by 18%) in 45-day-old NDM rats compared to the control (Figure 3B). In contrast, in PDM rats, the TGC-ANV was reduced at both ages was more pronounced on day 45 (significantly by 25%) than on day 25 (insignificantly by 20%).

The data for TGC-ANV were expressed relatively to the Sertoli cell volume as the ratio indicates the efficiency of Sertoli cell support for spermatogenesis, in particular, how many germ cells are supported by one Sertoli cell. The mean values of this parameter in NDM (12.99 ± 1.25) and PDM (14.02 ± 0.85) were nearly that of the control value (13.30 ± 2.17).

**Figure 4:**
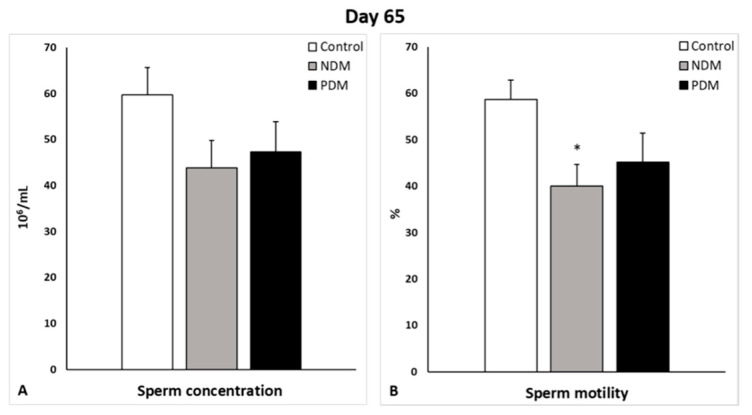
Sperm concentration (10^6^/mL, (**A**)) and motility (%, (**B**)) of 65-day-old control and diabetic rats. Data represent the mean value ± standard error (SE); * *p* < 0.05—statistical significance compared to the control value. NDM—neonatally-induced diabetes; PDM—prepubertally-induced diabetes.

On day 65, sperm concentration and sperm motility were evaluated on spermatozoa isolated from both cauda epididymides of the control and diabetic rats. Sperm concentration was lower by 20% and 25% in the PDM and NDM than the control, respectively. A similar decrease was established for sperm motility (%)—by 20% and 30% in PDM and NDM, respectively. A high percentage of round-shaped undifferentiated cells was seen in NDM (11.48 ± 1.83) but not as much in PDM (4.11 ± 0.56), whereas in the control samples, they were 0.90 ± 0.13 (Figure 4).

### 3.5. Leydig Cell Counts and Measurements of Testosterone Levels

**Figure 5:**
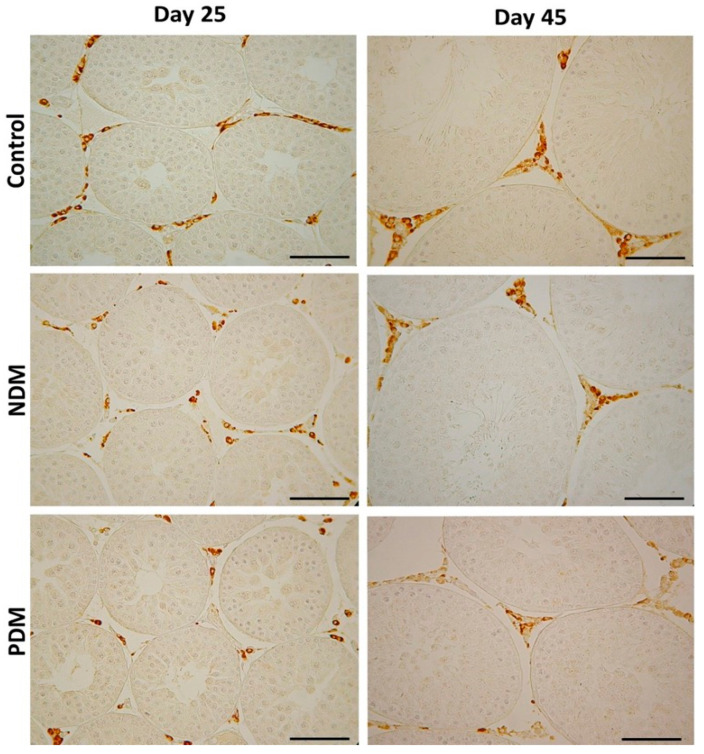
Immunohistochemical visualization with the marker enzyme 3β-hydroxysteroid dehydrogenase (3β-HSD) in Leydig cells in 25-day-old and 45-day-old control and diabetic rats with neonatal (NDM) and prepubertal diabetes (PDM). Scale bar = 50 µm.

Leydig cell counts were performed after visualization by the specific marker enzyme 3β-hydroxysteroid dehydrogenase (Figure 5). The enzyme catalyzes the conversion of Δ5-3β-hydoxysteroids to Δ4-3-ketosteroids, thus is considered as a key enzyme in androgen biosynthesis.

**Figure 6:**
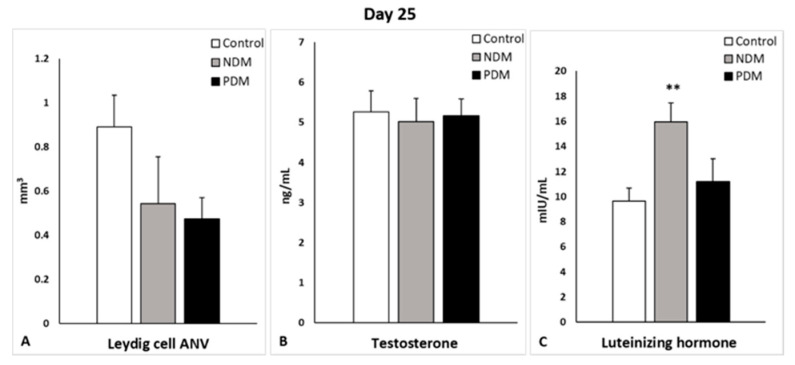
Absolute nuclear volume (ANV) of Leydig cells (mm^3^) (**A**), serum testosterone levels (ng/mL) (**B**), and serum luteinizing hormone (mlU/mL) (**C**) on day 25 of control and diabetic rats. ** *p* < 0.01—statistical significance compared to the control value. NDM—neonatally-induced diabetes; PDM—prepubertally-induced diabetes.

**Figure 7:**
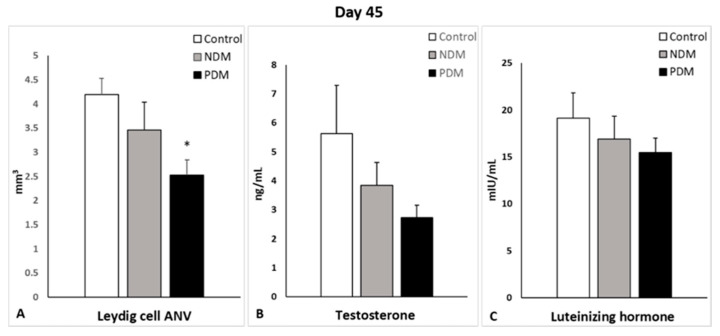
Absolute nuclear volume (ANV) of Leydig cells (mm^3^) (**A**), serum testosterone levels (ng/mL) (**B**) and serum luteinizing hormone (mlU/mL) (**C**) on day 45 of control and diabetic rats. * *p* < 0.05—statistical significance compared to the control value. NDM—neonatally-induced diabetes; PDM—prepubertally-induced diabetes.

Quantification of Leydig cells in the testicular interstitium on day 25 and day 45 revealed lower ANV (LC-ANV) in both diabetic groups, and reached statistical significance in 45-day-old PDM rats. The tendency of decrease in LC-ANV was more pronounced in the PDM group compared to NDM and was similar in PDM rats aged 25 and 45 days when compared to the control (by 55–60%) (Figure 6A and Figure 7A).

Serum testosterone concentrations were not changed in the 25-day-old NDM and PDM groups (Figure 6B). However, the tendency of decrease in testosterone levels on day 45 was 31.5% in NDM and 51% in PDM rats compared to the controls (Figure 7B). Statistical significance was reached in the adult 65-day-old PDM group (decrease by 63.5%).

In order to explain the normal testosterone levels in 25-day-old diabetic groups where LC-ANV was reduced, we calculated the ratio of the absolute nuclear to cytoplasm volume of Leydig cells. Interestingly, the ratio was higher by 13% in the NDM (3.15 ± 0.42) and by 37% in PDM (3.84 ± 0.68) groups compared to the control (2.80 ± 0.58).

Serum LH concentrations were elevated in both pubertal groups (aged 25 days) compared to the control, which was more pronounced in NDM (by 65%) than PDM (by 16%) (Figure 6C). In post-pubertal rats (aged 45 days), the hormone levels were insignificantly lower by 12% and 20% in NDM and PDM, respectively (Figure 7C).

### 3.6. Quantification of the Protein Expression of Androgen Receptor in the Testis

**Figure 8:**
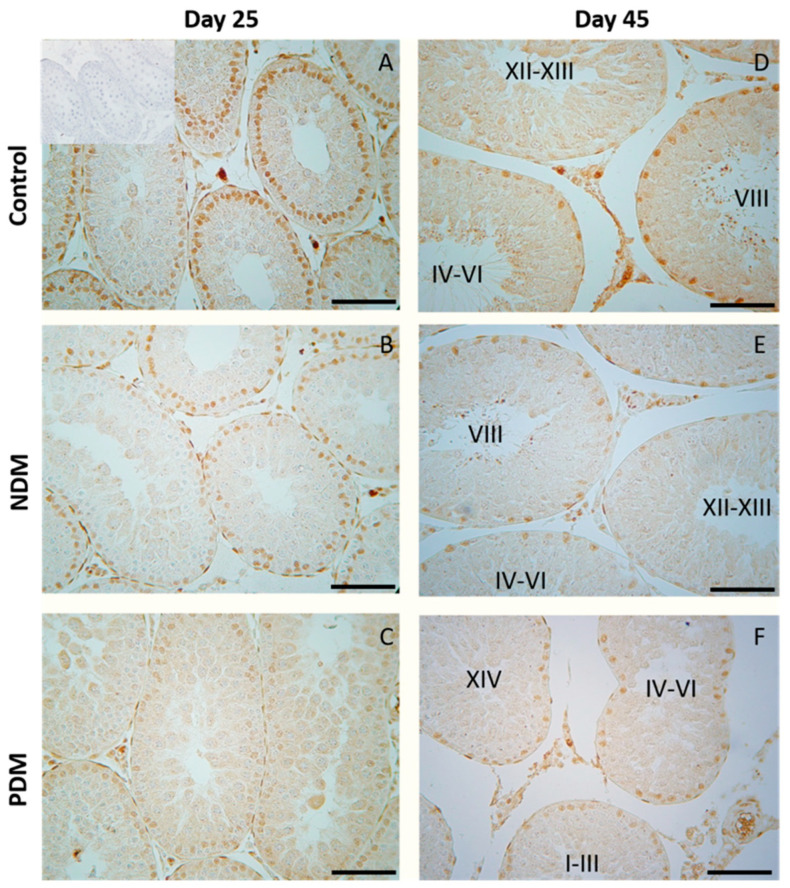
Immunohistochemical expression of androgen receptor (AR) in Sertoli cells in 25- and 45-day-old control (**A**,**D**) and rats with neonatal DM (**B**,**E**) and prepubertal DM (**C**,**F**). A weaker intensity of reaction was observed in Sertoli cells of PDM rats. Note the uniform intensity of the reaction in Sertoli cells of PDM rats aged 25 and 45 days. Scale bar = 50 µm. Negative control was carried out by pre-absorption of the primary antibody with peptide immunogen (picture inserted into the control picture of day 25).

**Figure 9:**
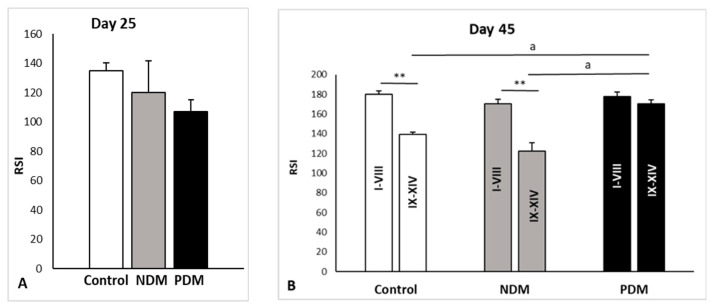
Measurement of androgen receptor (AR) immunostaining in Sertoli cell nuclei of 25- (**A**) and 45-day-old (**B**) rat testes using ImageJ. On day 45, the intensity of AR immunostaining is presented for stages I–VIII and late stages (IX–XIV) for each experimental group. The values in graphs are shown as reciprocal staining intensity (RSI), where RSI = 255—mean gray value. Data represent the mean value ± standard error (SE); ** *p* < 0.01—statistical significance compared to the control value. a—statistical significance between groups. NDM—neonatally-induced diabetes; PDM—prepubertally-induced diabetes.

In the pubertal control testis (day 25), when spermatogenesis was not completed, the stage specificity of AR protein expression in Sertoli cells was not pronounced (Figure 8A). NDM did not produce any significant changes in AR protein expression in Sertoli cells (Figure 8B,C and Figure 9A).

In 45-day-old control rats, androgen receptor was localized in the nuclei of Sertoli cells, Leydig cells, and peritubular cells but not in germ cells. As we previously reported, the stage-specific protein expression of AR was evident in the nuclei of Sertoli cells through the fourteen stages of the spermatogenic cycle in adult rats [22]. Intense expression was seen at early stages (I–VI, RSI = 175 ± 5.5), with the maximum at stages VII–VIII (RSI = 185 ± 2.4) of the cycle while the lowest expression was found at stages IX–XIV (RSI = 139 ± 9.5) (Figure 8D and Figure 9B). Quantitative measurements of androgen receptor immunostaining in the SC nuclei of the control testes showed significant differences when comparing the late stages to early or middle stages (VII–VIII) (Figure 9B). The same pattern was observed in NDM animals in the early (I–VI) stages, RSI = 165 ± 4.9, middle (VII–VIII), RSI = 174 ± 8.3, and the late (IX–XIV) stages, RSI = 123 ± 6.5. Due to incomplete spermatogenic cycle in 45-day-old PDM rats where a partial or complete lack of elongating spermatids in stages I–VIII was established, it was not possible to distinguish the early (I–VI) from middle (VII–VIII) stages. For this reason, in PDM animals, a comparison in the intensity of AR immunostaining was performed between stages I–VIII and the late stages (IX–XIV). Differences between experimental groups were also evaluated by a comparison of the intensity of AR immunostaining between stages I–VIII and late stages (IX–XIV). In the control rats, there were significant differences between AR immunostaining in stages I–VIII and late stages (IX–XIV) that were not established in the PDM animals (Figure 9B). As a result, the stage specificity of AR expression was not evident on 45-day-old PDM rats where uniform immunoreactivity in the tubules from all stages was found—the RSI of stages I–VIII was 178 ± 4.1, and the RSI of PDM stages IX–XIV was 170 ± 3.7 (Figure 8F and Figure 9B).

## 4. Discussion

During the last two decades, although a large number of studies both on diabetic men and experimental diabetic animals have been published about the impact of DM on male reproduction, many of them have conflicting results. The prevailing notion is that DM alters spermatogenesis, sperm parameters, biosynthesis of testosterone and induces degenerative changes in the testis, which lead to sub-fertility or infertility. Although extensive research has been conducted on experimental models for the induction of DM in adulthood (sexually maturity), studies on the effect of hyperglycemia on immature animals are very limited.

The early postnatal period is known to be critical for the development of male germ cells, where after a prolonged resting period in fetal and neonatal life, the precursors of germ cells (pre-spermatogonia) resume mitotic division on day 4.5 in rats, giving rise to differentiated spermatogonia. The latter proceed to a series of consecutive divisions to enter the first prophase of meiotic division with the formation of primary spermatocytes occurring on day 12 in rats. Both major events are crucial for the establishment of spermatogenesis, and any interference during this time results in poor reproductive capacity and infertility [17]. Aside from the initiative role of pituitary gonadotrophic hormones and androgen locally produced in the testis, insulin might also have a direct action on testicular cell composition. Germ cells are highly dependent on carbohydrates needed for energy homeostasis, being vulnerable to any disturbance in glucose metabolism [12,23].

By evaluating the impact of DM induced in early life, the current study provides new data suggesting that neonatal or prepubertally induced hyperglycemia might exert differential effects on testicular cell populations (germ and somatic cells) during the first wave of spermatogenesis, with a possible impact on semen quality.

Glucose levels, as measured on day 25, showed that the NDM rats were normoglycemic, as expected [see King, 2012 [5] for neonatally induced T2DM], and later developed hyperglycemia. PDM animals were hyperglycemic on day 25 and then maintained their DM status until days 45 and 65 in tandem with insulin insufficiency, suggesting that PDM rats might represent the T1DM model. Our unpublished data on insulin resistance index support that NDM rats developed T2DM, exhibiting slightly decreased insulin levels and insulin resistance in contrast to PDM, which were insulin deficient and non-resistant.

In our model, 25-day-old control and experimental rats were deprived of food for 2 h, and for this reason, we considered their glucose was non-fasting as it was not according to the standard protocol for overnight fasting [24]. According to Carper et al. [25], 2 h of fasting is optimal to assess insulin tolerance in rodents (mice and rats), which have a faster metabolic rate than humans.

Day 25 in rats is considered as mid-puberty, and spermatogenesis proceeds to late pachytene-diplotene spermatocytes. Day 45 represents post-puberty/early adulthood as spermatogenesis is completed but without sperm ejaculation, and therefore animals are still sexually immature.

Quantification of total germ cell population on day 25 and day 45 revealed different effects of both types of DM. NDM resulted in a slight elevation of TGC-ANV at both ages by 9–18% compared to the controls while PDM caused a decrease in this parameter by app. 25% than the control. Limited data are available where the germ cell number was reduced in adult animals with T1DM or T2DM induced by different treatment protocols (high fat diet or nicotinamide plus STZ as well drinking of fructose) [19,26,27,28,29]. In the few papers published on neonatally-induced DM, there are no data on testicular cell numbers. A comparative study by Barsiah et al. [26] demonstrated a more pronounced reduction in germ and Sertoli cell number by T1DM than adult T2DM. Our new data for germ cell counts suggest different effects on spermatogenesis caused by both types of DM—PDM reduced TGC-ANV in contrast to NDM, which did not produce a negative effect.

Evaluation of the proceeding of spermatogenesis in post-pubertal diabetic rats (45-day-old) demonstrated, for the first time, a delayed development of the late stages in spermatogenesis (elongated spermatids, visualized by tACE), in PDM but not in NDM rats. Quantification of the STs with altered tACE phenotype in PDM rats provided new evidence about the pattern of delayed spermatid development. The initial step of spermatid elongation, which occurs in late stages, was less altered compared to more advanced steps in early stages (I–VI). The STs in stages IV–VI were the most affected as none of them contained elongated spermatids. These data are indicative of the delayed completion of spermatogenesis due to impaired late steps of spermatid elongation. It seems that germ cells are more vulnerable to PDM than to NDM, which could be explained by different glucose profiles and different administration times of STZ. PDM was induced at the time of the active proliferation of spermatogonia before entering meiosis, while NDM was induced during the quiescent period (mitotic arrest) of precursor spermatogonia (pre-spermatogonia).

Many studies have indicated that apoptosis and oxidative stress, in tandem with inflammation, are responsible for germ cell loss in diabetic conditions (summarized in review articles) [12,13,14]. Recently, we reported the increased protein expression of pro-apoptotic factor Bax, which was more pronounced in the PDM than NDM rats, and these data could provide an explanation for the decreased TGC-ANV in PDM animals [30]. Moreover, the levels of some molecular markers for oxidative stress [3-nitrotyrosine (3-NT) and 4-hydroxynonenal (4-HNE)] were more elevated in PDM than in NDM, which was associated with an increased protein expression of the pro-inflammatory marker, tumor necrosis factor alpha (TNF-α) [31].

To follow the consequences of impaired spermatogenesis on fertility, we evaluated the semen parameters (sperm count/concentration and sperm motility) on day 65 in both diabetic groups. The sperm count and sperm motility decreased to a similar extent in PDM and NDM. An extremely high number of round shaped abnormal undifferentiated cells were counted (11-fold increase) in NDM but not in PDM, which might be a reason for the decreased sperm parameters in NDM despite no obvious destructive changes in the testes of post-pubertal 45-day-old rats. Most papers have reported a decreased sperm concentration and motility in T1DM [29,32,33,34] and in T2DM [27], but data from NDM in adulthood are contradictory—decreased semen parameters [16] vs. increased sperm concentration [35].

There is a general agreement that changes in absolute testis weight reflect those in TGC-ANV, and our results support this finding. Most of the data in the literature indicate lower absolute testis weight in adult rodents with T1DM and T2DM. Barsiah et al. [26] reported decreased testis weight only in adult animals with T1DM but not in T2DM—a finding that fits our results regarding the different effect of PDM and NDM on the testis weight. We found higher absolute volumes of seminiferous epithelium (SE) and tubular lumen in 45-day-old NDM rats in contrast to lower values in PDM. There are discrepancies in the literature data regarding the testicular macro-parameters due to different treatment regimens. Some studies have reported a decrease in SE volume, ST, and lumen diameter in diabetic rats [19], while other investigations found an elevation in their values [26,33].

Sertoli cells are essential for developing germ cells to sustain spermatogenesis by providing them with a unique microenvironment, physical support, growth factors, and appropriate nutrients including glucose. Any metabolic alteration in these cells caused by DM might be responsible for impaired spermatogenesis, resulting in compromised fertility. These cells have glucose sensing machinery that reacts to hormonal fluctuations, and several mechanisms operate to counteract hyper/hypoglycemic events [23]. Extensive research has been conducted on the expression of molecular markers for oxidative stress and inflammation including innate immune response under the condition of hyperglycemia [11,36]. Less data are available on quantitative aspects of adult Sertoli cells in conditions of DM, but none are available for Sertoli cells of developing testis. Our data revealed that at pubertal age, NDM and PDM did not produce any significant changes in SC-ANV. A reduced number of SCs and GCs was reported in adult T1DM and T2DM associated with increased apoptosis [19,27,29,37]. A possible explanation for the slight elevation in SC-ANV in NDM post-pubertal rats in our study might be explained by the higher absolute testis weight. Increased Sertoli cell number was demonstrated by Tavares et al. [38] in in vitro studies on neonatal mouse organ cultures as well on the TM4 Sertoli cell line treated by D-glucose. An important indicator for Sertoli cell support toward germ cells is the ratio of TGC-ANV–SC-ANV [18], and in the current study, we did not find any evidence for altered Sertoli cell supportive function.

Sertoli cells have long been considered the prime candidates for the androgen regulation of spermatogenesis because of the specific expression of AR through the stages of seminiferous epithelium. The current study did not find any significant changes in AR expression in Sertoli cells in pubertal 25-day-old NDM and PDM rats. Later, at the post-pubertal stage when spermatogenesis was completed, the stage-specific pattern in Sertoli cells was not maintained in PDM. Instead, a uniform pattern of AR expression was seen as a result of increased expression in the late stages of the spermatogenic cycle. Such a phenomenon has been reported by us under other experimental conditions of hormonal manipulation such as androgen withdrawal [22]. A possible compensatory mechanism of androgen signaling could be suggested, as it is essential for developing germ cells under the condition of compromised androgen production in PDM. According to Ballester et al. [39], AR protein levels (measured by Western blot) were not changed in T1DM adult rats while Favaro et al. [40] found a reduced expression of AR in prostate epithelium (reduced number of AR positive cells and protein levels in tissue homogenates). Our results on the quantification of AR protein expression provide new knowledge on altered androgen action in post-pubertal Sertoli cells by PDM but not by NDM. Such a finding might explain reduced TCG-ANV as a result of the delayed development of advanced stages of spermatogenesis (elongated spermatids).

Leydig cells are known to drive spermatogenesis via the synthesis and secretion of testosterone after stimulation by pituitary luteinizing hormone. Testosterone, in turn, acts on Sertoli cells to establish a unique environment for the normal progression of germ cells through the spermatogenic cycle [8]. Experimental manipulations for androgen deficiency have provided evidence for a reduced size of the adult Leydig cell population [18]. After visualization by a specific LC marker (3β-HSD), we found decreased LC-ANV in pubertal and post-pubertal NDM and PDM rats compared to controls that was more pronounced in PDM. Intact testosterone production in both pubertal DM groups could be explained by an increased ratio of nuclear to cytoplasm volume, indicative that LCs are functionally more active in conditions of hyperglycemia. The elevation in LH levels in PDM on day 25 could be interpreted as a compensatory mechanism for providing stimuli to the LCs so that they can maintain normal testosterone production. At the end of puberty, the LC-ANV and testosterone production of LCs were less influenced by NDM compared to PDM, where the changes in testosterone concentration corresponded to that in LC-ANV. Our results for low levels of LH reduced the testosterone concentration on day 45, which is suggestive of a lack of a classical feedback mechanism. Young patients with type 2 diabetes have significantly lower plasma testosterone concentrations and “inappropriately” low LH and FSH concentrations with a very high prevalence of hypogonadotrophic hypogonadism [41]. Data for decreased levels of testosterone, LH, and FSH in human males and in animal models were summarized by Maresch et al. (2018) [4] and have also been reported in adult rats with alloxan-induced T1DM and T2DM (by fructose drink) [24]. Our finding for reduced insulin concentrations in 45-day-old PDM could explain the lower level of LH and testosterone. Decreased testosterone and LH levels in adult diabetic rats resulted from the suppression of insulin, which was able to adversely affect Leydig cell proliferation and hormone production [37,39].

There are some discrepancies between the data on LC number and T biosynthesis in T1DM and T2DM diabetic models. Most of the studies demonstrated decreased number of LCs as well as reduced serum T levels [19,29,37,39]. In other studies, Leydig cell hypertrophy or hyperplasia and decreased T levels were reported [15,42]. In addition, low levels of testosterone and pituitary gonadotropic hormones (FSH and LH) were summarized in recent review articles [12,13,43]. Downregulation of the expression of key genes of androgen biosynthesis is involved in the suppression of testosterone production in Leydig cells [44,45]. One paper reported different hormonal profiles between adult T1DM and T2DM [26]—decreased serum gonadotrophins in T1DM but not in T2DM. These data support our results, suggesting that the ANV of adult LCs and their testosterone production is affected by PDM and altered to a lesser extent by NDM.

## 5. Conclusions

Taking into account the limitation of rodent models in extrapolating the findings to human males, we developed a rat experimental model of DM bearing in mind the common mechanisms in mammals that are involved in androgen dependent/driven events in the establishment of spermatogenesis. For this purpose, we applied a new approach for the comparative evaluation of the development of testicular cell populations under the condition of hyperglycemia induced at different time points of early postnatal development when two crucial events for the establishment of spermatogenesis occur. NDM and PDM models in developing rats provide new data for the differential response of testicular cell types (germ cells, Sertoli cells and Leydig cells) to hyperglycemia depending on the time of its induction (neonatally or prepubertally). Moreover, differential response of the testis is related to different glucose and insulin profiles in developing animals. However, PDM and NDM may have similar effects on some end points like relative testis weight, germ cell/Sertoli cell ratio, sperm concentration, and motility. PDM exerted a negative impact on germ cell development and the proceedings of spermatogenesis. The LC-ANV and testosterone levels were reduced in tandem with compromised expression of AR in Sertoli cells. Thus, our data provide new insights into the mechanism of action of early DM on developing germ cells that involve disturbances in androgen production by Leydig cells and androgen action in Sertoli cells. Suggesting that PDM rats developed T1DM in adulthood and NDM—T2DM, our DM models could be useful for future research into diabetes-related male infertility. Our data on young DM animals during the development of hyperglycemia might be related to pre-diabetic status, which is interesting from a clinical point of view, so monitoring is also recommended for pre-diabetic patients. We hope that our data for the differential impact of both type of DM could have clinical relevance for a more precise approach to reduce the negative impact of diabetes mellitus on the reproductive health of patients.

## Figures and Tables

**Table 1:** Serum glucose levels (mmol/L) in control and diabetic animals on days 25 (non-fasting), 45, and 65 (fasting).

	Day 25	Day 45	Day 65
Control	10.86 ± 0.50	7.22 ± 0.84	5.55 ± 0.25
NDM	11.49 ± 0.54 (6% ↑)	9.04 ± 1.27 (27% ↑)	10.02 ± 0.62 ** (80% ↑)
PDM	14.00 ± 1.80 (30% ↑)	13.73 ± 3.72 * (93% ↑)	10.00 ± 1.22 ** (80% ↑)

Data are presented as the mean ± standard error (SE); * *p* < 0.05; ** *p* < 0.01—statistical significance compared to the control value; NDM—neonatally-induced diabetes; PDM—prepubertally-induced diabetes. Arrows show elevation of glucose levels compared to control.

**Table 2:** Comparison of the absolute volumes of the seminiferous epithelium, lumen, and interstitial tissue in the control and diabetic groups (NDM and PDM) on days 25 and 45.

		Interstitium (mm^3^)	Lumen (mm^3^)	Seminiferous Epithelium (mm^3^)
Day 25	Control	31.32 ± 2.32	14.09 ± 2.61	136.36 ± 10.39
	NDM	28.03 ± 4.47	13.53 ± 2.47	131.11 ± 12.29
	PDM	20.45 ± 2.52	8.30 ± 2.07 *	105.25 ± 10.07
Day 45	Control	116.27 ± 14.11	47.87 ± 7.74	637.86 ± 35.54
	NDM	108.41 ± 6.62	105.73 ± 30.76	756.86 ± 34.30
	PDM	96.38 ± 6.47	40.89 ± 10.22	478.73 ± 40.36 *

Data represent the mean value ± standard error (SE); * *p* < 0.05—statistical significance compared to the control value. NDM—neonatally-induced diabetes; PDM—prepubertally-induced diabetes.

## Data Availability

All data needed to understand this study are included in the manuscript.

## Abbreviations

The following abbreviations are used in this manuscript:

DM	Diabetes mellitus
T1DM	Type 1 diabetes mellitus
T2DM	Type 2 diabetes mellitus
STZ	Streptozotocin
DNA	Deoxyribonucleic acid
AR	Androgen receptor
T	Testosterone
GCs	Germ cells
FSH	Follicle-stimulating hormone
NDM	Neonatally induced diabetes mellitus
PDM	Prepubertally induced diabetes mellitus
i.p.	Intraperitoneal
b.w.	Body weight
ELISA	Enzyme-linked immunosorbent assay
IHC	Immunohistochemistry
3β-HSD	3β-hydroxysteroid dehydrogenase
tACE	Testicular angiotensin converting enzyme
TBS	Tris buffered saline
BSA	Bovine serum albumin
ROI	Regions of interest
RSI	Reciprocal staining intensity
HEPES	N-2-Hydroxyethylpiperazine-N-2-ethane sulfonic acid
ANV	Absolute nuclear volume
LC	Leydig cell
LH	Luteinizing hormone
HFD	High fat diet
NAD	Nicotinamide adenine dinucleotide
GC-ANV	Germ cell absolute nuclear volume
TGC-ANV	Total germ cell absolute nuclear volume
3-NT	3-Nitrotyrosine
4-HNE	4-Hydroxynonenal
TNF-α	Tumor necrosis factor α
SE	Seminiferous epithelium
ST	Seminiferous tubules
SC	Sertoli cell
